# Self‐nudging toward physical activity: Scale development, validation, and workplace implications

**DOI:** 10.1111/aphw.70129

**Published:** 2026-02-11

**Authors:** Arnold B. Bakker, Hiroyuki Toyama, Jumpei Yajima, Suguru Iwano, Lauri Hietajärvi, Katja Upadyaya, Katariina Salmela‐Aro

**Affiliations:** ^1^ Center of Excellence for Positive Organizational Psychology Erasmus University Rotterdam Rotterdam The Netherlands; ^2^ School of Management, Human Resource Management University of Vaasa Vaasa Finland; ^3^ School of Economics and Business University of Ljubljana Ljubljana Slovenia; ^4^ Department of Industrial Psychology and People Management University of Johannesburg Johannesburg South Africa; ^5^ Department of Psychology Lingnan University Hong Kong Hong Kong; ^6^ Department of Education University of Helsinki Helsinki Finland; ^7^ Department of Human Studies University of Beppu Beppu Japan; ^8^ Center for Medical Education and Career Development Fukushima Medical University Fukushima Japan

**Keywords:** nudging theory, physical activity, self‐nudging, self‐regulation, work engagement

## Abstract

This study uses nudging theory to develop and validate a measure for self‐nudging toward physical activity. The research unfolds in three phases: (a) initial item development through interviews and literature review, (b) psychometric testing with Japanese employees (*N* = 1540), and (c) validity assessment in a longitudinal subsample (*N* = 716). Factor analyses confirm a reliable one‐factor structure. The scale demonstrates content and predictive validity, showing positive relationships with physical activity, physical capacity (fitness and energy), well‐being (workability, work engagement, and job satisfaction), and job performance; and negative relationships with strain (psychological distress, exhaustion, and occupational depression). Structural equation analyses show that self‐nudging at Time 1 is positively related to well‐being and negatively related to strain at Time 2 (3 months later) through increased physical capacity, with effects remaining significant after controlling for baseline measures. The findings contribute to our understanding of the associations among self‐nudging, physical activity, and occupational well‐being. The results have implications for both theoretical advancement in nudging research and practical applications in workplace health promotion.

## INTRODUCTION

Despite the compelling evidence showing the health benefits of regular physical activity—including reduced risks of cardiovascular disease, osteoporotic fractures, and dementia, as well as improved weight management, cognition, and subjective well‐being—many adults fail to meet recommended physical activity levels (Buecker et al., 2021; Conn et al., 2011; Daskalopoulou et al., 2017). Because the sedentary nature of many jobs necessitates prolonged sitting and minimal physical activity (Gardner et al., 2016), there is a strong need for workplace interventions that promote movement and combat health risks. Unfortunately, reviews and meta‐analyses suggest only small positive effects of workplace interventions to facilitate physical activity (e.g., Chu et al., 2016; Conn et al., 2011; Freak‐Poli et al., 2020; Lusa et al., 2020). Despite the use of sit–stand desks and activity trackers, or participation in organized activities, such as walking, cycling, and standing intervals, employees often struggle to maintain their physical activity routines over time.

In the present study, we investigate whether people can use self‐nudging to regulate their physical activity. Self‐nudging is a form of self‐leadership (Unsworth & Mason, 2012) that involves using behavioral strategies to motivate oneself to engage in more physical activity and reduce sedentary behavior. The concept builds on the principles of social nudging (Thaler & Sunstein, 2008)—a behavioral economics theory built on the premise that decisions are often based on intuitive rather than thoughtful processes (Kahneman, 2017). The central proposition of nudge theory is that by altering the environment (i.e., the *choice architecture*) in a way that influences behavior predictably without restricting options or significantly changing economic incentives, nudges can guide individuals toward making better decisions and achieving desired outcomes.

When self‐nudging, individuals create their own choice architecture to guide their decisions toward healthier behaviors (Reijula & Hertwig, 2022). Self‐nudging toward physical activity may include creating visual cues, setting up reminders to be physically active, establishing incentives or rewards for meeting activity goals, or modifying the environment to make physical activity more convenient and appealing (To & Bakker, 2018). In contrast with social nudging, self‐nudging gives individuals control over their own default choices without external influence and allows them to tailor nudges to their specific preferences and needs (Tontrup & Sprigman, 2022).

We aim to make four contributions to the literature. Based on nudging and proactivity theories, we first develop the construct of self‐nudging toward physical activity. We address the lack of a standardized measure for studying self‐nudging, bridging this gap to enable future research to systematically investigate self‐nudging practices. Second, we argue and show that self‐nudging is predictive of physical activity and is positively related to fitness and energy. This provides the necessary validation of the measure and will demonstrate the relevance of self‐nudging as a behavioral strategy to motivate oneself to engage in more physical activity. Third, we argue that self‐nudging has advantages for well‐being and for functioning at work—we focus on psychological distress, work‐related exhaustion, occupational depression, work ability, work engagement, job satisfaction, and job performance. By exploring the nomological net of self‐nudging, we further test its construct and predictive validity. Fourth and finally, we build self‐nudging theory by proposing and testing indirect favorable effects of self‐nudging toward physical activity. We propose that self‐nudging increases fitness and energy, and that these physical resources can, in turn, be used to increase well‐being and work‐related outcomes.

## THEORETICAL BACKGROUND

Traditionally, health behavior change policies and interventions have focused on reflective and conscious processes, that is, providing people with information, seeking to change the way people think about their behavior, or providing (financial or legal) incentives that change the consequences of behavior (Cecchini et al., 2010). Unfortunately, these traditional approaches are only moderately effective (Rongen et al., 2013; Vlaev et al., 2016). One possible reason is that our behavior is also determined by automatic and unconscious processes that have largely been ignored in the health behavior change literature. Nudge theory (Thaler & Sunstein, 2008) proposes that individuals often use heuristics to simplify decision‐making. Accordingly, individuals are greatly influenced by the environment within which decisions are made (Vlaev et al., 2016). By using knowledge about cognitive biases and intuitive decision‐making, we can design environments (i.e., the choice architecture) in ways that facilitate health behavior (Thaler & Sunstein, 2008; Volpp et al., 2008).

In a recent review, Forberger et al. (2022) identified 26 interventions aimed at promoting physical activity or reducing sedentary behaviors in the workplace using nudges. In most studies advocating physical activity, participants were encouraged to use stair climbing. This is carried out, for example, by placing arrows on the floor to guide individuals toward using the stairs instead of elevators, or by designing staircases to resemble a piano keyboard where each step produces a musical note when stepped on, making stair climbing a fun activity. Only 54% of the nudge interventions were effective, showing effects on stair use, walking, and (reduced) sitting time. Factors contributing to the ineffectiveness of social nudges may include diminished autonomy because of perceived paternalism (“why do others restrict my freedom of choice?”), psychological reactance (Brehm & Brehm, 2013) and the inherent limitations of social nudges in accommodating the diverse needs and preferences of larger populations (De Ridder et al., 2024; Dewies et al., 2021).

### Self‐nudging

Self‐nudging refers to employee proactive behaviors aimed at changing the personal choice architecture such that health behaviors are more likely. This form of nudging uses a blend of slow, thoughtful processes (called System 2) and fast, intuitive processes (System 1) to guide behavior effectively (Kahneman, 2017). Self‐nudging implies that individuals first think consciously about how they can nudge themselves by taking the personal initiative to create idiosyncratic nudges. According to Tontrup and Sprigman (2022), people who use self‐nudges should recognize and understand their present bias or decisional errors they aim to overcome, while also proactively anticipating that modifying their decision‐making environment will effectively harness the bias they seek to leverage. Self‐nudging initiatives to be physically active (using System 2) are, for example, downloading a smartphone app that tracks various activities and provides challenges, or the placement of dumbbells in the office so that the individual is regularly reminded to engage in health behaviors without conscious deliberation (using System 1). A study among real estate employees (To & Bakker, 2018) showed that individuals use various nudges that they initiate themselves daily. Participants who used self‐nudges were indeed more physically active and reported more energy. Reijula and Hertwig (2022) argue that self‐nudges require an understanding of how the environment influences behavior and the ability to apply specific nudges to shape this interaction for desired outcomes.

In this study, we develop a new instrument to assess self‐nudging toward physical activity. In Phase 1, we use interviews and the nudging literature to identify various nudging techniques that individuals could potentially use to increase physical activity. In Phase 2, we examine the factorial validity of an instrument including a selection of the self‐nudging items, as well as its test–retest reliability. In Phase 3, we investigate the construct and predictive validity of the self‐nudging scale.

## PHASE 1: ITEM DEVELOPMENT

The development of a new scale for self‐nudging toward physical activity followed a systematic yet creative approach. This process integrated structured interviews, targeted literature searches, and theoretical insights from behavioral economics and behavior change research to identify a broad and diverse range of strategies that individuals may use to promote their own physical activity. A total of 107 individuals were interviewed by a research assistant regarding their physical activity habits and the tools, reminders, or strategies they employ to support these behaviors. The interviews lasted 45 min on average. Although detailed demographic information was not collected—limiting the representativeness of the sample—it is known that 57% of the respondents identified as female. To initiate reflection and facilitate discussion, participants were first prompted with the following two questions: *“How physically active would you consider yourself?”* and *“How many times per week do you engage in these activities?”* These were followed by an open‐ended question: *“Do you use any tools, reminders, or strategies to increase the likelihood that you will follow through on being physically active?”*

In addition, a systematic literature search was conducted using PsycINFO, PubMed, and Google Scholar. The search employed the following keywords: “self‐imposed nudging,” “self‐initiated nudging,” “self‐nudging and health‐related habits,” and “reminder apps use and exercise.” All qualitative data were analyzed using ATLAS.ti (ATLAS.ti Scientific Software Development GmbH, 2023), a software tool that facilitates the systematic coding and interpretation of textual data. Thematic content with conceptual overlap was consolidated to reduce redundancy. This procedure resulted in a list of 37 distinct self‐nudging strategies. Although it is unlikely that this list exhaustively captures every possible nudge, it offers a rich and potentially comprehensive overview of strategies that individuals may realistically use to support their physical activity. Recognizing the need for a more concise and practically applicable instrument, we refined the list to 13 core self‐nudging items. These items were slightly adapted in wording to enhance generalizability and ensure relevance across different contexts and populations. The resulting set was designed to be tested in new samples to evaluate its psychometric properties and practical utility in behavioral research.

## PHASE 2: FACTORIAL VALIDITY

In the second phase, we investigate the factorial validity and reliability of the newly developed scale for the assessment of self‐nudging toward physical activity.

## METHOD

### Participants and procedure

Ethical clearance was obtained from the university's ethics committee prior to the commencement of the study. The participants were recruited through Cross Marketing Group Inc., a social survey service provider in Japan (https://www.the-cmj.com/). The first survey was conducted in September 2023. An invitation to participate in the survey was distributed to registered individuals (*N* = 35,652). The participants were allocated to be evenly distributed in terms of gender and age group. The survey could be accessed for 3 days, and 1540 employees responded to the survey within that period. After 3 months, these individuals were invited to the second survey. In total, 716 employees responded to the same survey—a retention rate of 46.5%.

The final sample at Time 1 consisted of 1540 workers, with 49.9% of them being male. The mean age of the sample was 45.1 years (*SD* = 13.5). More than half of the participants (56.4%) had a bachelor's degree or higher degrees. Of all, 47.3% was married. The occupational field of participants varied, including clerical work (34.7%), professional/technical (15.3%), sales and marketing (12.9%), services (10.6%), production skills and operations (8.0%), management (6.7%), transportation and communication (2.8%), security (0.5%), nature (0.3%), and others (8.2%). Most participants had a permanent contract (80.7%). The participants worked 9.4 h/day (*SD* = 4.4) on average. Of the total sample, 55.6% engaged in regular exercise, with an average frequency of 1.7 days/week. The mean duration of exercise was 0.7 h/week (*SD* = 1.1).

The final *panel* sample comprised 716 workers from various sectors. Half of the sample (50.6%) was male, and the mean age was 45.4 years (*SD* = 13.4). More than the half of the participants (55.3%) had a bachelor's degree or higher degrees, and 45.4% was married. The distribution of occupational category in this sample was very similar to the sample at Time 1: most common was clerical work (37.3%), followed by professional/technical (14.2%), sales and marketing (12.2%), services (10.2%), production skills and operations (7.4%), management (7.1%), transportation and communication (2.9%), security (0.7%), and nature (0.4%). An additional 7.5% was employed in other fields. The average working hours were 9.5 h/day (*SD* = 4.5). Most participants had a permanent contract (79.5%). Of the total sample, 54.2% engaged in regular exercise, averaging 1.7 days/week. The mean duration of exercise was 0.7 h/week (*SD* = 0.9).

We conducted chi‐square tests to examine whether dropout status was associated with key demographic and occupational variables. Gender distribution did not differ significantly between dropouts and completers, *χ* ^2^(1, *N* = 1540) = 0.21, *p* = .648. Similarly, no significant differences were found for educational background, *χ* ^2^(4, *N* = 1540) = 3.07, *p* = .546, or occupational category, *χ* ^2^(9, *N* = 1540) = 7.39, *p* = .597. Type of employment also showed no significant association with dropout status, *χ* ^2^(4, *N* = 1540) = 6.73, *p* = .151. These results indicate that attrition was not systematically related to participants' demographic characteristics or employment status.

### Measures

#### Self‐nudging

Employees' self‐initiated nudging toward physical activity was assessed using the self‐nudging items developed in Phase 1. The participants were requested to look back at the past month and indicate the extent to which they agreed with 13 statements such as “I used reminders to be physically active”, and “I made sure that had fitness equipment in sight for a short exercise break” (1 = s*trongly disagree, 7* = s*trongly agree*).

## RESULTS

We split the data into two random halves so that we could first conduct an exploratory factor analysis (EFA) on the first half of the sample (*N* = 770), and then a confirmatory factor analysis (CFA) on the second half (*N* = 770). The EFA using maximum likelihood extraction with varimax rotation yielded a two‐factor solution. The first factor accounted for 58.9% of the variance, while the second factor explained 12.2%. The Kaiser–Meyer–Olkin (KMO) Measure of Sampling Adequacy was 0.941—well within the “marvelous” range—indicating that the correlation matrix was highly factorable and that the observed variables shared sufficient common variance to warrant factor analysis. This strong KMO value supports the robustness of the factor solution and lends confidence to its interpretability. Despite both factors having eigenvalues greater than 1, closer inspection revealed that the items loading on the second factor did not adequately reflect the self‐nudging construct. Specifically, the three items—“I motivated myself to be physically active,” “I purposefully made exercise part of my routine,” and “I reminded myself to walk or use the stairs”—were conceptually broader and less targeted compared to the more nuanced items defining the first factor. These items also accounted for limited additional variance. Therefore, we excluded them from further analysis on theoretical and statistical grounds, retaining only those items that loaded cleanly onto a single, interpretable factor representing self‐nudging. We then used the data of the first half of the sample again to run a similar EFA using the 10 remaining items. This resulted in a clear one‐factor solution with the factor explaining 66.8% of the variance. All items loaded 0.45 or higher on the latent factor—in fact, nine of the 10 items had factor‐loadings higher than 0.73 (see Table 1, first column). The internal consistency of the scale was good, Cronbach's *α* = 0.94.

**TABLE 1:** Exploratory and confirmatory factor analytic results (factor‐loadings) for the self‐nudging toward physical activity scale.

	Self‐nudging item	EFA (T1, *N* = 770)	CFA (T1, *N* = 770)	CFA (T2, *N* = 716)
1	I used reminders to be physically active	0.83	0.83	0.82
2	I made sure that had fitness equipment in sight for a short exercise break	0.87	0.87	0.85
3	I proactively talked about my fitness goal with others	0.86	0.86	0.86
4	I proactively shared my physical activity with friends through social media	0.86	0.86	0.85
5	I proactively adjusted my work so that I could be physically active	0.83	0.83	0.80
6	My smartphone reminded me of physical activity goals throughout the workday	0.82	0.82	0.81
7	I set up my smartphone to track my steps/activities.	0.45	0.45	0.41
8	I used wearables (e.g., fitness tracker, smart watch) to track my physical activity	0.74	0.74	0.69
9	I actively monitored the benefits of physical exercise	0.79	0.79	0.75
10	I designed health cues for myself (e.g., running shoes in sight, hand weights on my desk)	0.81	0.81	0.83

Abbreviations: CFA, confirmatory factor analysis; EFA, exploratory factor analysis; SD, standard deviation.

We next conducted a CFA on the second half of the sample (*N* = 770) using Amos 29.0 (Arbuckle, 2022). The initial one‐factor model showed acceptable but imperfect fit, *χ* ^2^(35) = 447.115, Goodness‐of‐Fit Index (GFI) = 0.882, Tucker–Lewis Index (TLI) = 0.915, Comparative Fit Index (CFI) = 0.934, and Root Mean Square Error of Approximation (RMSEA) = 0.124. To address localized misfit, four theory‐driven residual covariances were specified between adjacent or content‐overlapping items, capturing localized shared method variance while preserving local independence (Hayes & Usami, 2020; Kaplan, 1988). This resulted in a substantial improvement in model fit, *χ* ^2^(31) = 241.211, GFI = 0.940, TLI = 0.951, CFI = 0.966, RMSEA = 0.094, and Standardized Root Mean Square Residual (SRMR) = .030. Although RMSEA remained marginally above the conventional 0.08 threshold, this should be interpreted cautiously given the model's low degrees of freedom (*df* = 31), as RMSEA is known to overestimate misfit in low‐df models (Kenny et al., 2015). In contrast, CFI and TLI exceeded the recommended 0.95 benchmark, and SRMR indicated excellent absolute fit (Goretzko et al., 2023; McNeish & Wolf, 2023; Ximénez et al., 2022). All factor loadings exceeded 0.45 in the unmodified solution (see Table 1), and internal consistency was high (Cronbach's *α* = 0.94). Taken together, these results support the adequacy of the measurement model, while warranting replication in an independent sample.

Finally, we conducted a CFA on the second‐wave data of all participants who also filled out the self‐nudging scale 3 months later (*N* = 716). Also at Time 2, the one‐factor model fit reasonably well to the data, χ^2^(35) = 407.049, GFI = 0.89, TLI = 0.911, CFI = 0.930, but again the RMSEA was high with a value of 0.122. Again, the specification of four theory‐driven residual covariances between adjacent or content‐overlapping items resulted in improved model fit, *χ* ^2^(31) = 183.503, GFI = 0.951, TLI = 0.959, CFI = 0.971, RMSEA = 0.083, SRMR = 0.041. All factor loadings were higher than 0.41, and Cronbach's *α* = 0.93. Additionally, the test–retest reliability, assessed over a 3‐month period, showed moderate stability (*r* = .67, *p* < .001) (cf. Nunnally & Bernstein, 1994). Taken together, these findings offer evidence for the factorial validity and reliability of the self‐nudging scale. The scale demonstrates a robust unidimensional structure and exhibits high internal consistency and reasonable test–retest reliability.

## PHASE 3: CONVERGENT AND PREDICTIVE VALIDITY

After securing the factorial validity of the self‐nudging scale, we continue by investigating its construct and predictive validity. Although self‐nudging is a technique used to facilitate fast decision‐making, it does imply that individuals first consciously design personalized strategies to influence their own behavior, creating tailored interventions that align with their unique goals and preferences. According to Tontrup and Sprigman (2022), people who self‐nudge must possess self‐awareness, identifying their cognitive biases and decision‐making flaws, while proactively crafting environmental modifications that capitalize on these tendencies to guide them toward desired outcomes—in this study: physical activity.

People with proactive personalities are more likely to use self‐nudging techniques. A proactive personality is characterized by a consistent tendency to initiate changes in one's environment and take action across various situations. This trait reflects an individual's natural inclination to be proactive (Bateman & Crant, 1993). Given this tendency, we can reasonably expect that these individuals will be more inclined to adopt self‐nudging strategies, which involve actively shaping one's environment to support physical activity.

Hypothesis 1 Proactive personality is positively related to self‐nudging.

One other important variable in the nomological network is obviously physical activity. Successful implementation of the self‐nudging strategy is expected to result in an increase in physical activity levels (e.g., exercise, walking, stair climbing, cycling, and running). The nudges serve to prompt individuals to stay active by automating the decision‐making process, effectively reminding them to engage in physical movement effortlessly. Previous research has shown that nudges can have a positive impact on physical activity, although the effects were relatively small (Forberger et al., 2022). Self‐nudging is hypothesized to be more effective because individuals actively select the strategies that suit them best. Rather than being externally customized, the nudging process is inherently tailored through self‐selection, with the individual acting as the agent who identifies and applies the nudges most relevant to their own needs and preferences.

Hypothesis 2 Self‐nudging is positively related to physical activity.

Furthermore, self‐nudging is expected to lead to higher levels of physical fitness and energy because of its influence on physical activity. Physical fitness has been defined as “the ability to carry out daily tasks with vigor and alertness, without undue fatigue and with ample energy to enjoy leisure‐time pursuits and to meet unforeseen emergencies” (Caspersen et al., 1985). By using self‐nudges, individuals can overcome biases and make more favorable decisions regarding their physical activity levels. By providing gentle reminders and incentives to engage in active behaviors, self‐nudges empower individuals to make incremental changes that cumulatively contribute to improved cardiorespiratory and muscular endurance, muscular strength, body composition, and flexibility (e.g., Bahls et al., 2021; Leblanc et al., 2015). Additionally, self‐nudging can help individuals develop healthier habits (Buecker et al., 2021; Reijula & Hertwig, 2022). Therefore, we propose:

Hypothesis 3 Self‐nudging is positively related to (a) fitness and (b) energy.

Self‐nudging toward physical activity is expected to have beneficial effects on psychological distress, work‐related exhaustion, and occupational depression. Physical activity functions as a recovery mechanism that promotes both psychological detachment from work and physiological restoration, thereby reducing stress responses and enhancing well‐being (Naczenski et al., 2017; Ten Brummelhuis & Bakker, 2012). Activities such as walking, running, dancing, and structured workouts facilitate mental disengagement from work‐related concerns and support mood regulation through neurobiological and psychosocial pathways (Rebar et al., 2015). These recovery processes are particularly relevant in reducing psychological distress (i.e., symptoms of anxiety, irritability, and emotional tension; Goldberg & Williams, 1988), work‐related exhaustion (i.e., chronic depletion of physical and mental energy caused by work demands; Schaufeli et al., 2020), and occupational depression (i.e., a clinically relevant form of low mood and loss of interest that is attributed to one's job context; Bianchi et al., 2023).

Crucially, these health‐promoting effects of physical activity are not limited to momentary relief but can translate into enduring psychological resources. One such resource is physical self‐efficacy, or the belief in one's ability to be active and physically competent (Joseph et al., 2014). This domain‐specific confidence can generalize to the work domain via resource gain spirals (Hobfoll et al., 2018), enhancing employees' belief that they can effectively manage job demands. Increased self‐efficacy has been linked to lower burnout and higher job performance (Bakker et al., 2023), suggesting that physical activity may have indirect effects on occupational health by enhancing individuals' perceived capability and resilience (Bandura, 1997; Rhee et al., 2017). We therefore hypothesize that

Hypothesis 4 Self‐nudging is negatively related to (a) psychological distress, (b) work‐related exhaustion, and (c) occupational depression.

Hypothesis 5 Self‐nudging is negatively related to strain (psychological distress, work‐related exhaustion and occupational depression) through physical capacity (fitness, energy).

Individuals who nudge themselves toward physical activity may do so throughout the day—at work as well as during leisure time. Such self‐nudging may also have an impact on positive, motivational work outcomes. Here we focus on workability (Van den Berg et al., 2009), work engagement (a positive, fulfilling state characterized by feelings of energy, dedication and enthusiasm about work, and absorption—full concentration on work activities; Schaufeli & Bakker, 2023), job satisfaction (a pleasurable emotional state resulting from the appraisal of one's job experiences; Locke, 1976), and job performance.

Physical activity has long been associated with enhanced energy regulation, improved cognitive functioning, and elevated mood—all of which are essential for sustained workplace effectiveness (Reed & Buck, 2009; Roig et al., 2013; Verburgh et al., 2014). When employees actively engage in strategies that help them regulate their energy—such as through structured breaks, light exercise, or movement reminders—they report higher levels of work engagement and job performance (Op den Kamp et al., 2018). Notably, a Finnish birth cohort study using objective accelerometer data demonstrated that greater physical activity and less sedentary behavior were associated with higher work engagement, underscoring the real‐world relevance of activity‐based energy regulation (Kiema‐Junes et al., 2022).

Self‐nudging—using behavioral prompts and cues to guide one's own behavior—serves as a practical tool for enhancing physical activity during the workday. Unlike external interventions, self‐nudging leverages intrinsic motivation and contextual awareness, enabling individuals to align behavioral strategies with their daily routines and energy needs (Hertwig & Grüne‐Yanoff, 2017). By promoting brief, intentional bouts of movement or exercise, self‐nudging can improve physical capacity—manifested as greater fitness, endurance, and vitality—which is a fundamental resource for sustained occupational functioning (Sonnentag & Fritz, 2007).

Enhanced physical capacity enables employees to invest more energy and effort into their work. For example, De Vries et al. (2022) discovered that employees who exercised during lunch breaks experienced a noticeable boost in momentary vigor—characterized by heightened cognitive liveliness and emotional energy—immediately afterward. While the study did not confirm a mediation effect, vigor following the lunch break positively influenced creative work performance in the afternoon. Greater fitness and energy allow employees to work with stamina, sustain attention, and remain resilient throughout the day (Erickson et al., 2019; Puetz, 2006)—key drivers of workability, engagement, job satisfaction, and ultimately job performance (Bakker & van Wingerden, 2021; Shirom, 2010). Thus, self‐nudging may function as an upstream strategy that is associated with greater occupational well‐being and productivity, potentially through its link to improved physical resources.

Hypothesis 6 Self‐nudging is positively related to (a) workability, (b) work engagement, (c) job satisfaction, and (d) job performance.

Hypothesis 7 Self‐nudging is positively related to well‐being (workability, work engagement, and job satisfaction) through physical capacity (fitness and energy).

## METHOD

### Participants and procedure

For the description of the samples, we refer to Phase 2. Next to the self‐nudging scale, the participants filled out questionnaires to assess proactive personality, physical activity, fitness, energy, psychological distress, work exhaustion, occupational depression, work engagement, job satisfaction, workability, and job performance. We used the cross‐sectional (*N* = 1570) and longitudinal data (*N* = 716) to test the hypotheses.

Consistent with our demographic analyses, we examined dropout patterns across all study variables to assess selective attrition. The participants who completed both waves (*n* = 716) were compared to those who dropped out after Time 1 (*n* = 824). Dropouts reported significantly higher psychological distress (*M* = 2.21, *SD* = 0.51) than completers (*M* = 2.15, *SD* = 0.52), *t*[1538] = 2.36, *p* = .018, *d* = 0.12. Similar effects were observed for work exhaustion (*t*[1538] = 2.28, *p* = .023, *d* = 0.12) and occupational depression (*t*[1538] = 2.20, *p* = .028, *d* = 0.11). No significant differences emerged for self‐nudging, physical activity, fitness, or energy (all *ps* > .05), indicating that attrition was unrelated to the study's core predictor variables. Additionally, dropouts did not differ significantly from completers on proactive personality (*p* = .407), work engagement (*p* = .255), or job satisfaction (*p* = .811). However, dropouts reported slightly lower workability (*t*[1538] = −1.97, *p* = .049, *d* = −0.10) and job performance (*t*[1538] = −3.03, *p* = .002, *d* = −0.15). Taken together, these results suggest that attrition was primarily related to psychological strain rather than differences in the study's primary constructs. As a result, the Time 2 sample may underrepresent individuals with elevated stress, potentially leading to conservative estimates of associations involving strain‐related variables.

### Measures

#### Proactive personality

The six‐item version of the Proactive Personality Scale (Bateman & Crant, 1993; Claes et al., 2005) was used to assess employees' proactive personality at Time 1. An example item is “No matter what the odds, if I believe in something I will make it happen”. Items could be scored on a 5‐point Likert scale ranging from 1 (*not at all true*) to 5 (*very true*). The reliability of the scale was good, with Cronbach's *α* = 0.84.

*Physical activity* was assessed with a single item: “To what extent do you engage in physical activities done alone (e.g., walking, cycling, swimming, strength training)?” Responses were given on a 4‐point scale ranging from 1 = “*I don't do it*” to 4 = “*More than twice a week*.” While the use of a single‐item measure is a limitation, the item is direct and easy to interpret, minimizing subjectivity. Prior research supports the reliability and validity of single‐item physical activity measures, showing strong test–retest correlations (*r* = .72–.82) and moderate concordance with more comprehensive tools such as the Global Physical Activity Questionnaire and the UK Active People Survey (Milton et al., 2011).

*Fitness* was measured by the five‐item International Fitness Scale (Ortega et al., 2011). The participants rated their own current fitness (relative to their friends) using a 5‐point Likert scale ranging from 1 (*very poor*) to 5 (*very good*). Example items are the following: “My cardiorespiratory fitness is …”, “My muscular strength is …”, and “My speed/agility is …”. The reliability of this scale was high, Cronbach's α = 0.94 at Time 1 and *α* = 0.95 at Time 2.

#### Energy

Employees' energy level was assessed by the 3‐item Circadian Energy Scale (Ottoni et al., 2011). An example is “During the past month, how was your energy level in the morning at work?” The participants rated their level of energy for morning, afternoon, and evening on a 5‐point Likert scale ranging from 1 (*very low*) to 5 (*very high*). Cronbach's *α* = 0.85 (Time 1) and *α* = 0.89 (Time 2).

#### Psychological distress

We used the General Health Questionnaire (GHQ)‐12 (Goldberg & Williams, 1988) to measure employees' psychological distress. The GHQ‐12 is a screening tool designed to detect non‐psychotic psychiatric morbidity. This scale is widely used in occupational health research to assess employees' general psychological health. The participants indicated whether they had experienced listed 12 symptoms of psychological distress over the last few weeks (e.g., “Have you recently lost much sleep over worry?”) using a 4‐point Likert scale from 0 to 3, with higher scores indicating higher levels of psychological distress. Cronbach's *α* = 0.87 and 0.88.

*Work exhaustion* was measured using the three‐item subscale of the Burnout Assessment Tool (Schaufeli et al., 2020). The items are, “At work, I feel mentally exhausted,” “After a day at work, I find it hard to recover my energy,” and “At work, I feel physically exhausted.” The participants rated how often they experienced these symptoms using a frequency scale ranging from 1 (*never*) to 5 (*always*). Cronbach's *α* was 0.94 at both timepoints.

#### Occupational depression

The 10‐item occupational depression inventory (Bianchi & Schonfeld, 2020) was used to measure employees' depressive symptoms stemming from work. An example item is “I felt depressed because of my job.” The participants rated how often they experienced each symptom over the last two weeks using a 4‐point frequency scale ranging from 0 (*never or almost never*) to 3 (*nearly every day*). Cronbach's *α* was 0.92 at both timepoints.

#### Work ability

Perceived work ability was assessed by a single item from the Work Ability Index (WAI): “How many points would you give your current ability to work?” Previous research has shown that this item accurately reflects the total WAI instrument (Jääskeläinen et al., 2016). The scoring options ranged from 0 (*cannot currently work at all*) to 10 (*work ability at its lifetime best*).

#### Work engagement

Work engagement was assessed using the 9‐item version of the Utrecht Work Engagement Scale (Schaufeli et al., 2006). This instrument is the most frequently used instrument to assess employee work engagement, and its validity was demonstrated in previous research (Schaufeli & Bakker, 2023). Example items are “At my job, I feel strong and vigorous” (vigor), “I am enthusiastic about my job” (dedication), and “I am immersed in my work” (absorption). The participants rated the items on a 7‐point scale ranging from 0 (*never*) to 6 (*always*). Cronbach's *α* was 0.97 at both time points.

*Job satisfaction* was assessed using a single‐item measure adapted from Van den Broeck et al. (2010): “How satisfied were you with your job during the past month?” This item was rated on a scale ranging from 1 (*very dissatisfied*) to 10 (*very satisfied*).

*Job performance* was measured using three items indicating in‐role behaviors adapted from Williams and Anderson (1991). The participants were instructed as follows: “Look back and evaluate your work performance over the past month,” and then responded to three statements, including “I fulfilled the responsibilities specified in my job description.” (1 = *strongly disagree*, 5 = *strongly agree*). Cronbach's *α* = 0.96 at both time points.

#### Strategy of analyses

Relationships between self‐nudging and other variables were tested using correlational analyses (Hypotheses 1–4 and 6). We tested the two mediation hypotheses (Hypotheses 5 and 7) using structural equation modeling (SEM), implemented through Amos software version 29.0 (Arbuckle, 2022). To maintain simplicity, we evaluated two separate models. In the first model, self‐nudging acted as the latent predictor variable with two indicators (even vs. uneven items), *change* in physical capacity was included as the mediator with two indicators (standardized residuals of fitness and energy), and T2 strain was the outcome with three indicators (psychological distress, work‐related exhaustion, and occupational depression). In this model, we controlled for the effect of T1 strain. In the second model, we used the same predictor and mediator, but now used T2 well‐being as the outcome with three indicators (workability, work engagement, and job satisfaction). Here, we controlled for the effect of T1 well‐being. In addition to testing the structural pathways, we employed the bootstrapping method with 5000 resamples using maximum likelihood estimation to test the proposed mediation effects.

## RESULTS

### Descriptive statistics

The means, standard deviations, and correlations between the study variables are presented in Table 2.

**TABLE 2:** Means, standard deviations, and intercorrelations for all study variables.

	Variables	Mean	*SD*	1	2	3	4	5	6	7	8	9	10	11	12	13	14	15	16	17	18	19	20	21	22	23
	Time 1																									
1	Proactive personality	3.01	0.7																							
2	Self‐nudging	2.39	1.34	.34***																						
3	Physical activity	2.17	1.28	.20***	.35***																					
4	Fitness	2.7	0.68	.36***	.33***	.25***																				
5	Energy	2.81	0.69	.44***	.32***	.19***	.51***																			
6	Psychological distress	2.18	0.51	−.29***	−.12***	−.13***	−.48***	−.48***																		
7	Work exhaustion	2.56	1.12	−.11***	−.07*	−.06*	−.34***	−.39***	.61***																	
8	Occupational depression	1.65	0.68	−.14***	−0.01	−0.04	−.32***	−.36***	.65***	.63***																
9	Workability	7.77	2.01	.28***	.07**	.11***	.41***	.40***	−.49***	−.35***	−.39***															
10	Work engagement	3.09	1.27	.51***	.35***	.20***	.41***	.54***	−.44***	−.32***	−.31***	.45***														
11	Job satisfaction	6.1	2.09	.30***	.15***	.13***	.40***	.45***	−.58***	−.46***	−.46***	.64***	.55***													
12	Job performance	3.43	0.89	.34***	.04	.11***	.32***	.39***	−.43***	−.24***	−.36***	.42***	.37***	.55***												
	Time 2																									
13	Proactive personality	3.02	0.69	.59***	.30***	.19***	.30***	.37***	−.27***	−.11**	−.17***	.22***	.40***	.25***	.29***											
14	Self‐nudging	2.69	1.29	.31***	.71***	.43***	.27***	.31***	−.14***	−.10**	−.05	.05	.29***	.10**	.04	.38***										
15	Physical activity	2.13	1.29	.17***	.44***	.72***	.21***	.17***	−.07	−.07	−.06	.06	.16***	.11**	.11**	.20***	.48***									
16	Fitness	2.74	0.7	.32***	.32***	.25***	.64***	.49***	−.46***	−.36***	−.34***	.41***	.41***	.40***	.26***	.35***	.29***	.25***								
17	Energy	2.83	0.69	.36***	.29***	.18***	.47***	.60***	−.49***	−.40***	−.40***	.41***	.50***	.44***	.35***	.44***	.31***	.21***	.56***							
18	Psychological distress	2.15	0.52	−.33***	−.13***	−.07	−.43***	−.49***	.75***	.58***	.61***	−.50***	−.48***	−.57***	−.41***	−.30***	−.17***	−.09*	−.51***	−.57***						
19	Work exhaustion	2.47	1.12	−.16***	−.06	−.02	−.33***	−.43***	.58***	.71***	.59***	−.36***	−.32***	−.42***	−.24***	−.14***	−.07	−.05	−.37***	−.45***	.62***					
20	Occupational depression	1.63	0.66	−.17***	−.04	−.04	−.34***	−.39***	.63***	.61***	.75***	−.45***	−.34***	−.48***	−.32***	−.13***	−.07	−.06	−.38***	−.41***	.69***	.66***				
21	Workability	7.87	1.97	.27***	.07	.12**	.42***	.41***	−.52***	−.40***	−.45***	.60***	.46***	.56***	.40***	.27***	.10**	.10**	.44***	.45***	−.54***	−.41***	−.48***			
22	Work engagement	3.11	1.24	.46***	.28***	.20***	.41***	.53***	−.45***	−.34***	−.32***	.41***	.80***	.53***	.37***	.45***	.35***	.18***	.44***	.56***	−.50***	−.33***	−.35***	.46***		
23	Job satisfaction	6.2	2.12	.30***	.14***	.03	.38***	.45***	−.55***	−.43***	−.47***	.52***	.54***	.68***	.45***	.29***	.16***	.08*	.44***	.50***	−.63***	−.45***	−.53***	.61***	.55***	
24	Job performance	3.52	0.87	.27***	.03	.07	.26***	.28***	−.37***	−.20***	−.32***	.36***	.37***	.44***	.54***	.28***	.04	.06	.30***	.30***	−.43***	−.20***	−.34***	.42***	.38***	.52***

*Note*: Time 1: *N* = 1570. Time 2: *N* = 716.

* *p* < .05.

** *p* < .01.

*** *p* < .001.

### Hypotheses testing

According to hypothesis 1, proactive personality is positively related to self‐nudging. Table 2 shows that the correlation between proactive personality and Time 1 self‐nudging (*r* = .34, *p* < .001) and T2 self‐nudging (*r* = .29, *p* < .001) is both significant. This means that individuals who are naturally inclined to take initiative are more likely to nudge themselves to be physically active. Hypothesis 1 is supported.

Hypothesis 2 states that self‐nudging is positively related to physical activity. As can be seen in Table 2, the participants who used more self‐nudging were more likely to be physically active during leisure time—engaging in personal activities, such as walking, running, and swimming, *r* = .35, *p* < .001. In addition, self‐nudging was positively related to Time 2 physical activity, *r* = .33, *p* < .001. These findings offer support for Hypothesis 2.

Hypothesis 3 states that self‐nudging is positively related to (a) fitness and (b) energy. Table 2 shows that self‐nudging to be physically active was positively related to fitness and energy within time (*r* = .33 and *r* = .32, respectively, both *p*'s < .001) and over time (*r* = .27, *r* = .25, both *p*'s < .001). These results support Hypothesis 3. Employees who frequently employ self‐nudging techniques tend to develop greater physical capacity, enhancing both their fitness levels and overall energy.

Hypothesis 4 proposes that self‐nudging to be physically active is negatively related to (a) psychological distress, (b) work‐related exhaustion, and (c) occupational depression. The results indicate that self‐nudging has a weak negative relationship with psychological distress. Specifically, the correlations with psychological distress, work‐related exhaustion, and occupational depression are as follows: at T1, *r* = −.12, *p* < .001; *r* = −.07, *p* = .011; and *r* = −.01, *p* = .634. The correlations with the T2 variables are *r* = −.13, *p* < .001; *r* = −.06, *p* = .123; and *r* = −.04, *p* = .296, respectively. These results provide partial support for Hypotheses 4a and 4b, while Hypothesis 4c is not supported by the data.

Hypothesis 6 states that self‐nudging is positively related to (a) workability, (b) work engagement, (c) job satisfaction, and (d) job performance. As can be seen in Table 3, self‐nudging to be physically active is particularly positively related to work engagement and job satisfaction. The correlations at T1 are *r* = .07, *p* = .006 (workability), *r* = .35, *p* < .001 (work engagement), *r* = .15, *p* < .001 (job satisfaction), and *r* = .04, *p* = .089 (job performance). The correlations with the T2 variables are *r* = .07, *p* = .059 (workability); *r* = .28, *p* < .001 (work engagement); *r* = .14, *p* < .001 (job satisfaction); and *r* = .03, *p* = .421 (job performance). Thus, our analysis yields mixed results: Hypothesis 5a is partially supported, Hypotheses 5b and 5c are fully supported, while Hypothesis 5d is not supported by the data.

**FIGURE 1:**
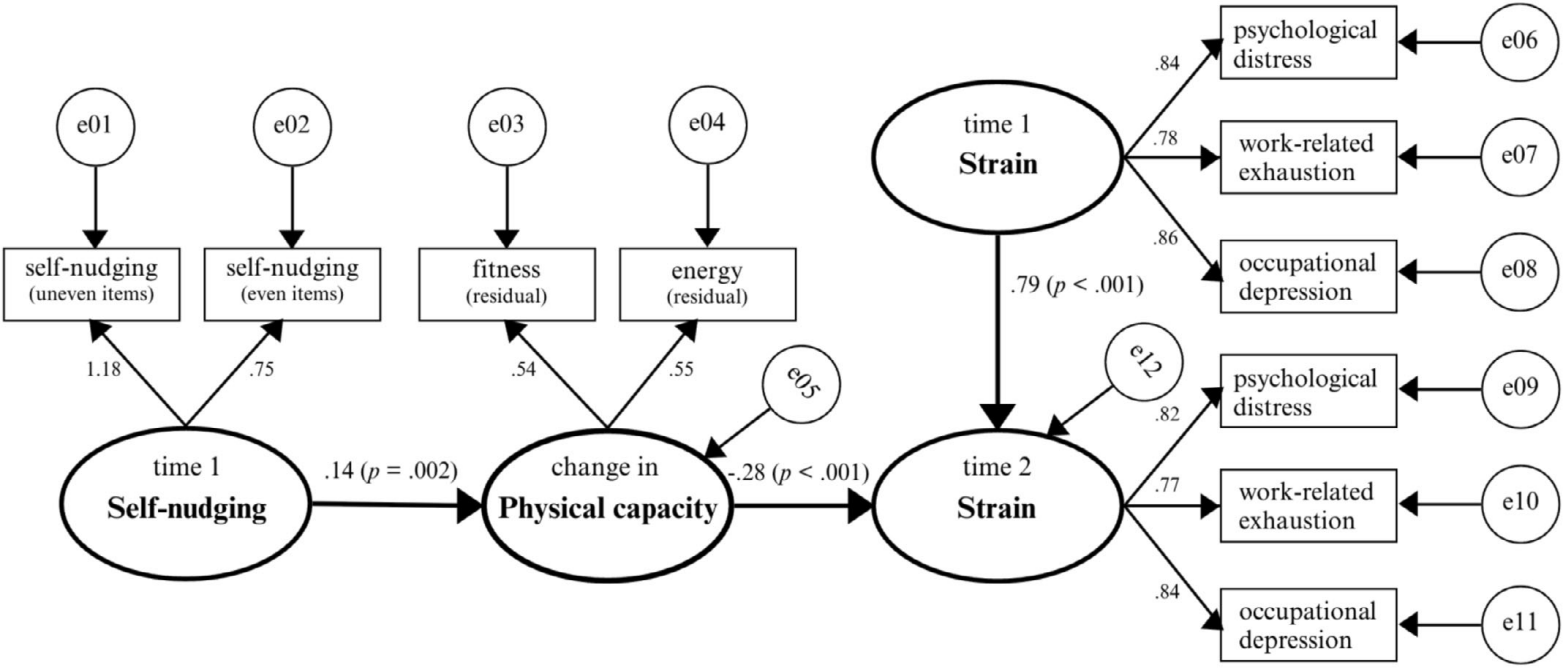
Results for self‐nudging and strain model based on maximum likelihood estimates (*N* = 716). *Note.* To keep the figure clear, some of the connections between predictors and between strain indicators over time are not shown.

Hypothesis 5 proposed that self‐nudging is indirectly negatively related to T2 strain via changes in physical capacity, controlling for T1 strain. The structural equation model shown in Figure 1 demonstrated good fit to the data, *χ* ^2^(27) = 73.425, *p* < .001, GFI = 0.980, RMSEA = 0.049, TLI = 0.982, CFI = 0.989, Incremental Fit Index (IFI) = 0.989. Results showed that self‐nudging significantly predicted improvements in physical capacity (*γ* = 0.14, *p* = .002). In turn, increased physical capacity was significantly negatively associated with T2 strain after controlling for T1 strain (*β* = −0.28, *p* < .001). The indirect effect of self‐nudging on T2 strain via physical capacity was also significant (*β* = −0.016, SE = 0.005, 95% CI = [−0.026, −0.007], *p* < .05), as bootstrapped confidence intervals excluded zero. These findings support the mediating role of physical capacity, suggesting that self‐nudging is negatively related to psychological distress, exhaustion, and depression through physical capacity. Thus, Hypothesis 5 was supported.

**FIGURE 2:**
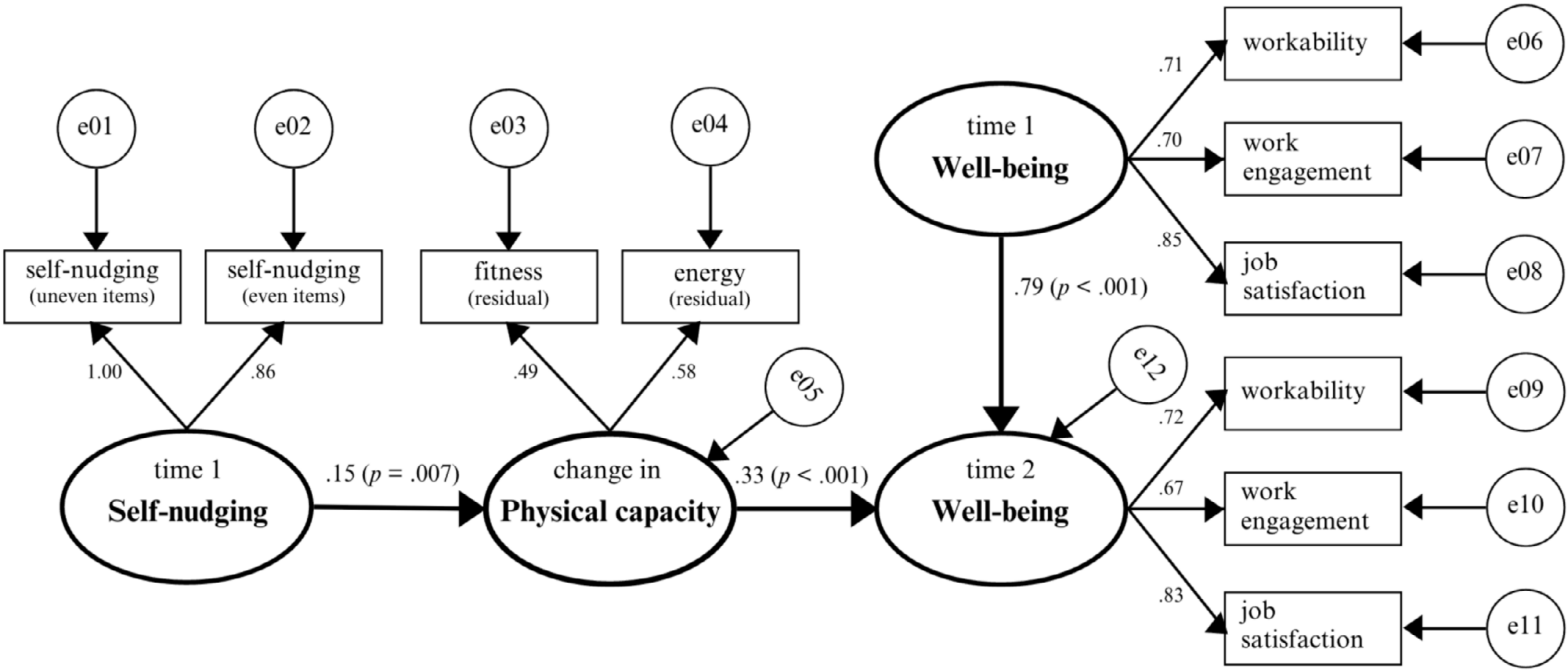
Results for self‐nudging and well‐being model based on maximum likelihood estimates (*N* = 716). *Note.* To keep the figure clear, some of the connections between predictors and between well‐being indicators over time are not shown.

Hypothesis 7 posited that self‐nudging is indirectly positively related to T2 well‐being via changes in physical capacity, controlling for T1 well‐being. Again, the model (Figure 2) fit the data well, *χ* ^2^(27) = 113.609, *p* < .001, GFI = 0.970, RMSEA = 0.067, TLI = 0.962, CFI = 0.977, and IFI = 0.977. Self‐nudging significantly predicted improvements in physical capacity (*γ* = 0.15, *p* = .007). Improved physical capacity was positively associated with T2 well‐being after controlling for T1 well‐being (*β* = 0.33, *p* < .001). The indirect effect of self‐nudging on T2 well‐being was also significant (*β* = 0.051, SE = .023, 95% CI = [0.012, 0.094], *p* = .031). These results suggest that self‐nudging is associated with higher Time 2 workability, work engagement and job satisfaction—potentially through its relation to physical capacity—providing support for Hypothesis 7.

## DISCUSSION

The central aim of the present study was to develop and validate a new instrument for the assessment of self‐nudging toward physical activity. The scale demonstrated strong psychometric properties. Results showed that self‐nudging toward physical activity is captured by one overall factor indicated by items that reflect social accountability, technological support, and behavioral activation. Thus, a variety of self‐nudging strategies could be captured by one overall factor that was first found with exploratory factor analysis and then replicated with CFA. Moreover, the one‐factor model was cross‐validated at T2 using the data of employees who participated in a second wave of data collection. The scale exhibited excellent reliability indicating that the construct of self‐nudging can be reliably assessed and has some stability over 3 months of time. While further validation across diverse cultural contexts is necessary, the new self‐nudging scale may spark further research into promoting physical activity, shedding light on how self‐nudging strategies work across different groups and settings. Future studies can explore where these strategies are most effective and where they might fall short, helping to refine our understanding of self‐nudging in health behavior change.

A second contribution of the present research is that we showed that individuals with a proactive personality are somewhat more likely to use self‐nudging. The correlation was moderate, and this is consistent with nudge theory (Mertens et al., 2022; Thaler & Sunstein, 2008). Accordingly, instead of thoughtful processes and plans, individuals often use heuristics to simplify decision‐making—they are influenced by the environment within which decisions are made (Vlaev et al., 2016). Our findings suggest that proactive employees who strategically modify their surroundings—through methods such as incorporating visual health prompts, openly discussing fitness objectives, and leveraging technology like smartphones and wearables for reminders—effectively streamline their decision‐making process for physical activity. This optimization of the environment reduced the need for conscious deliberation, making active choices more automatic and less reliant on proactive personality and willpower (cf. Thaler & Sunstein, 2008; Volpp et al., 2008).

Third, we found that self‐nudging is predictive of physical activity and seems to benefit fitness and energy. These findings underscore the construct validity of the measure and indicate that self‐nudging is an important behavioral strategy to motivate oneself to engage in more physical activity. This is the more important because there is compelling evidence for the health benefits of regular physical activity (e.g., Buecker et al., 2021; Daskalopoulou et al., 2017). Because the sedentary nature of many jobs necessitates prolonged sitting and minimal physical activity (Gardner et al., 2016), there is a strong need for interventions that promote movement and combat health risks among employees. The current study suggests that employees can use self‐nudging to be more active and increase their physical capacity (fitness and energy).

Moreover, the findings of the present study suggested that self‐nudging has several advantages for well‐being that extend to the work domain. Employees who used more self‐nudging experienced lower levels of general psychological distress, as well as lower levels of work‐related exhaustion and depression, because they had more physical capacity. The indirect effects on these strain outcomes were found after controlling for previous levels of strain, which provided a robust statistical test. It should be noted that previous research has clearly shown that high job demands, particularly when combined with low job resources, are important determinants of work‐related exhaustion (Bakker et al., 2023). If previous levels of strain (psychological distress, exhaustion, and occupational depression) can be taken as a proxy of the impact of working characteristics, then we see that self‐nudging has a unique and favorable impact. Self‐nudging seems to lower job strain because employees create physical capacity resources that can be used to deal with their job demands and a lack of job resources (cf. Bakker, 2015; Hobfoll et al., 2018).

Self‐nudging to be more physically active also predicted workability, work engagement, and job satisfaction—through physical capacity. These findings indicate that self‐nudging creates important energetic resources that are used in the work domain. These findings align with the work recovery literature, demonstrating that psychological detachment from work and relaxation during leisure time replenish energy and other volatile resources, enhancing work engagement and performance (Bennett et al., 2018; Sonnentag et al., 2022). However, self‐nudging distinguishes itself from recovery in a crucial way: while recovery is a *reactive* self‐regulation strategy aimed at *replenishing* depleted energy, self‐nudging serves as a future‐oriented approach, preserving fitness and energy levels, thereby safeguarding the individual's resource reservoir before depletion occurs.

It is interesting to note that self‐nudging had *indirect* effects on future well‐being and work‐related outcomes, but there were no clear *direct* effects on the non‐targeted outcomes. There may be two reasons for this. First, it makes sense that self‐nudging particularly has a direct impact on more proximal outcomes, such as physical activity and daily feelings of fitness and energy. According to the principle of correspondence (Ajzen & Timko, 1986), a predictor is more likely to correlate with an outcome if both variables show a strong correspondence (e.g., in terms of specific context, target, and timing). In the present study, the predictor self‐nudging was specifically directed toward daily physical activity in various contexts, whereas several outcomes, such as work engagement, refer to a very specific context (organizational) and a different target (work or job). Second, self‐nudging may be more indirectly than directly associated with well‐being and work‐related outcomes, as regular physical activity—often supported by self‐nudging—has been linked to improved cognitive functions such as memory, attention, and decision‐making (Roig et al., 2013; Verburgh et al., 2014). Thus, next to energetic resources, self‐nudging improves cognitive resources which then explain more distal outcomes. The present study showed that self‐nudging was indirectly related to various (work) outcomes, and that these effects were mediated by physical capacity.

Although social nudging has become popular and widely used in behavioral interventions, scholars have questioned its effectiveness because of the lack of strong, convincing evidence (Houdek, 2024; Maier et al., 2022). One major challenge is the complexity of human behavior. Individual differences and situational variability mean that a one‐size‐fits‐all approach rarely succeeds universally. Another issue is that there are many different types of nudges. When researchers look at all these different nudges together in large meta‐analytic studies, the results are mixed (Hummel & Maedche, 2019; Mertens et al., 2022). Some nudges work better than others, which can make the overall effect seem smaller. Moreover, people may perceive nudges as paternalistic, feeling their personal autonomy is being compromised (Hausman & Welch, 2010). This perception may lead to reactance—the psychological phenomenon where individuals resist influence attempts, potentially counteracting the intended effects of the nudge (Rosenberg & Siegel, 2018).

These constraints underscore the potential merits of *self*‐nudging as an alternative paradigm. By facilitating individual agency in environmental modification, self‐nudging offers the prospect of more tailored and context‐specific interventions, potentially circumventing limitations inherent in traditional social nudging approaches. The findings of the present study suggest that self‐nudging toward physical activity can be effective. Future research should aim to replicate these findings in various organizational settings, exploring how self‐nudging can promote other behaviors crucial for employee well‐being and organizational success. These may include healthy eating habits, improved sleep hygiene, stronger workplace social connections, effective stress management, and enhanced productivity. Understanding these relationships is vital for organizations seeking to create healthier, more resilient, and more productive work cultures, potentially leading to reduced health‐care costs, decreased absenteeism, and improved overall performance.

### Limitations and future research

Like all research, the present study has limitations. First, the findings can only be taken as preliminary evidence for the idea that self‐nudging can facilitate physical activity, capacity, and well‐being. The present study used a two‐wave design to test lagged mediation (see Figures 1 and 2). While this was a standard practice in earlier organizational research (Schaufeli et al., 2009; Ten Brummelhuis et al., 2011), recent methodological work recommends three or more waves with autoregressive paths to strengthen causal inference (Wang & Zhang, 2020; Zhang & Yang, 2020). Consequently, our evidence for mediation should be viewed as preliminary. Moreover, although we used a longitudinal research design to test indirect effects over time, reversed causal effects cannot be ruled out. To get more control over causality, future research may use an experimental design in which individuals *learn* how to develop self‐nudges to engage in desirable behaviors and where the effects are compared with a control group.

Second, we relied on self‐reported data which can be subject to biases such as social desirability or recall bias (e.g., Adams et al., 2005). The participants may have overestimated their use of self‐nudging, their physical activity, or their well‐being. To mitigate these biases, researchers may use experience sampling or diary study methodologies, wherein participants are monitored over extended periods, ranging from days to weeks. In this research, the participants regularly document their self‐nudging strategies, physical activities, and associated outcomes, providing a more dynamic and nuanced dataset. To enhance data validity, self‐reported measures could be complemented with objective physiological indicators (e.g., heart rate variability) and quantifiable metrics of physical exertion, such as accelerometer‐derived step counts and GPS‐based elevation data (e.g., Jeran et al., 2016).

Third, our self‐nudging findings are limited to the domain of physical activity. We developed a measurement instrument that specifically focuses on physical activity, while self‐nudging may be valuable for various other purposes, including fruit consumption, smartphone use, prosocial behaviors, and stress management (e.g., Grüning et al., 2023; Van Rookhuijzen et al., 2023). For example, self‐nudges toward prosocial behavior may include daily reminders to oneself to schedule time to explore opportunities to help colleagues, to provide help, or to become a volunteer. These specific behaviors were not part of the current instrument and study, and it remains to be seen whether more complex work behaviors can equally effectively be nudged as physical activity. Future research may use the current approach to test self‐nudging in different behavioral domains.

Fourth, individuals who prioritize physical health may be more likely to engage in self‐nudging strategies, whereas those with lower health motivation may use such strategies less consistently. Although our focus was on the general use of self‐nudging, future research could explore how individual differences in health orientation, exercise motivation, or attitudes toward physical activity shape daily self‐nudging behavior and its effectiveness. Fifth, although attrition was unrelated to demographics and key predictors, it was linked to psychological strain. As a result, the Time 2 sample may underrepresent individuals experiencing elevated stress, potentially leading to conservative estimates for strain‐related associations. Future research should aim to replicate these findings in samples with broader stress variability or lower dropout among high‐strain individuals.

Finally, our sample included Japanese employees, and thus it is unclear to what extent self‐nudging will also work for employees in other countries. In Japan, there is an increasing awareness of the need for exercise among workers (Matsuo & So, 2021). Many workers in Japan are becoming cognizant of a lack of exercise and wish to cultivate a consistent exercise routine. Therefore, it is possible that the present results reflect this unique Japanese culture. Future research should investigate the cross‐cultural applicability of our findings by examining workers in countries with diverse physical activity norms and exercise cultures.

### Practical implications

The present findings have important practical implications, but it would be advisable to first test the effects of a self‐nudging *intervention* to establish causality. Self‐nudging toward physical activity seems to improve fitness, energy, and (work‐related) well‐being, and thus organizations may want to encourage the use of self‐nudging by providing access to self‐nudges, including wearables, dumbbells, and the autonomy to regulate one's own physical activity (time control). Human resource managers could also organize self‐nudging workshops in which the concept of self‐nudging is explained, and where participants can practice developing their own idiosyncratic nudges. Moreover, individuals may collaborate in developing self‐nudging strategies, potentially becoming mutual motivators. This collaborative approach could manifest in various ways, such as partnering for physical activities during work breaks, collectively opting for stair use over elevators, coordinating gym visits, or engaging in joint training sessions. Such peer‐supported initiatives may enhance the effectiveness of self‐nudging by leveraging social dynamics and shared accountability.

### Conclusion

Our study introduces a reliable, one‐factor questionnaire for assessing self‐nudging, demonstrating its convergent and predictive validity. Results indicate that self‐nudging is associated with higher levels of physical activity, fitness, and energy, which are in turn linked to greater general and work‐related well‐being. This new measure offers a valuable tool for researchers and organizations alike, paving the way for deeper insights into self‐nudging strategies. By leveraging this tool, organizations can empower employees to improve their physical capacity and, consequently, their work engagement and job satisfaction. We hope our research will pave the way for innovative strategies that empower employees to thrive at work through the practice of self‐nudging.

## CONFLICT OF INTEREST STATEMENT

The authors declare no conflicts of interest with respect to the research, authorship, and/or publication of this article.

## ETHICS STATEMENT

This study was conducted in accordance with the ethical guidelines outlined in the Declaration of Helsinki. Ethical approval was obtained from the university's ethics committee prior to the commencement of the study. All participants provided informed consent prior to their participation in the study.

## Data Availability

The data that support the findings of this study are available from the corresponding author upon reasonable request.
